# Differential responses of chicken monocyte-derived dendritic cells infected with *Salmonella* Gallinarum and *Salmonella* Typhimurium

**DOI:** 10.1038/s41598-021-96527-w

**Published:** 2021-08-26

**Authors:** Degpal Singh, Mithilesh Singh, Vishal Chander, Gaurav Kumar Sharma, Manish Mahawar, Aamir Salam Teeli, Tapas Kumar Goswami

**Affiliations:** 1 Immunology Section, ICAR-Indian Veterinary Research Institute, Izatnagar, Bareilly, Uttar Pradesh 243122 India; 2 Centre for Animal Disease Research and Diagnosis, ICAR-Indian Veterinary Research Institute, Izatnagar, Bareilly, Uttar Pradesh 243122 India; 3 Division of Biochemistry, ICAR-Indian Veterinary Research Institute, Izatnagar, Bareilly, Uttar Pradesh 243122 India; 4 Institute of Genetics and Animal Biotechnology of the Polish Academy of Sciences, 05-552 Jastrzebiec, Poland

**Keywords:** Immunology, Microbiology

## Abstract

*Salmonella enterica* serovar Gallinarum is a host-restricted bacterial pathogen that causes a serious systemic disease exclusively in birds of all ages. *Salmonella enterica* serovar Typhimurium is a host-generalist serovar. Dendritic cells (DCs) are key antigen-presenting cells that play an important part in *Salmonella* host-restriction. We evaluated the differential response of chicken blood monocyte-derived dendritic cells (chMoDCs) exposed to *S.* Gallinarum or *S.* Typhimurium. *S*. Typhimurium was found to be more invasive while *S*. Gallinarum was more cytotoxic at the early phase of infection and later showed higher resistance against chMoDCs killing. *S*. Typhimurium promoted relatively higher upregulation of costimulatory and other immune function genes on chMoDCs in comparison to *S*. Gallinarum during early phase of infection (6 h) as analyzed by real-time PCR. Both *Salmonella* serovars strongly upregulated the proinflammatory transcripts, however, quantum was relatively narrower with *S*. Gallinarum. *S*. Typhimurium-infected chMoDCs promoted relatively higher proliferation of naïve T-cells in comparison to *S*. Gallinarum as assessed by mixed lymphocyte reaction. Our findings indicated that host restriction of *S*. Gallinarum to chicken is linked with its profound ability to interfere the DCs function. Present findings provide a valuable roadmap for future work aimed at improved vaccine strategies against this pathogen.

## Introduction

Salmonellae are facultative intracellular, anaerobic, rod-shaped, Gram-negative bacteria. They are of length 2–5 microns and width 0.5–1.5 microns. They are motile thanks to peritrichous flagella. *Salmonella enterica* subspecies *enterica* (*S. enterica*) is a member of the family Enterobacteriaceae, which has a very wide host range^1^. Enterobacteriaceae is divided into > 2600 serovars (a serovar is a distinct variation within a species of bacteria or virus). Most of these serovars are “host generalists” because they cause disease in several warm-blooded animals (e.g., mice, chicken, calves, humans)^2^. *S.* Enteritidis and *S.* Typhimurium are classical examples of the host-generalist serovars associated most frequently with foodborne infection^3,4^. In contrast, certain serovars restricted to one very specific host species are referred to as “host-restricted” or “host-specific” serovars^1^. Host-restricted serovars often cause systemic and fatal infections within their host (e.g., *S.* Typhi, *S.* Gallinarum, *S.* Abortusequi)^5^. *S.* Gallinarum causes a systemic fatal disease (fowl typhoid) in domestic birds (primarily chickens). Fowl typhoid seems to be endemic in many parts of the world.

There is a dearth of scientific knowledge regarding the factors and fundamental mechanisms contributing to the existence of host-restricted and host-generalist *Salmonella* serovars. Several microbial pathogens have evolved molecular mechanisms aimed at interfering with phagocytic cell (dendritic cells (DCs), macrophages) functions to allow survival within the host^6^. This is considered to be a hallmark of host-restriction for certain *Salmonella* serovars^7^.

*Salmonella* carries an array of effector molecules called the *Salmonella* pathogenicity island-2-encoded type-3 secretion system (SPI-2 T3SS), which facilitates intracellular survival in a specific host by modulating natural intracellular environments^7–9^. It is likely that antibodies and other humoral components may not have access to the intracellular *niche* which confers a “safe haven” for bacteria. *Salmonella* can survive within DCs, where it modulates the antigen-presenting capacity of these professional cells. Hence, interference with the ability of DCs to process and present bacterial antigens could be advantageous for *Salmonella* dissemination within a specific host. Some studies have suggested that *Salmonella* uses DCs as “vehicles” for transportation and dissemination to systemic sites in the host^10^. Studies have shown that host-restricted *Salmonella* serovars can interfere with the capacity of DCs to “prime” adaptive immunity against bacteria. In contrast, host-generalist *Salmonella* serovars could be unable to interfere with DC function, thus result in activation of the adaptive immune response and bacterial clearance^11,12^.

Considering the crucial part played by DCs in host-restriction and systemic dissemination of *Salmonella* serovars, the role of *S.* Gallinarum to affect DC function in chickens and, therefore, manage host-restriction, is not known.

We studied the in vitro interplay between host-restricted (*S.* Gallinarum) and host-generalist (*S.* Typhimurium) *Salmonella* serovars with chicken DCs. Our results suggested that the host specificity of *S.* Gallinarum may be related to its ability to interfere with DC function and, consequently, weak induction of the adaptive immune response.

## Methods

### Ethical approval of the study protocol

The study protocol was approved by the Animal Ethics Committee of Indian Veterinary Research Institute as per the guidelines of the Committee for the Purpose of Control and Supervision of Experiments on Animals (CPCSEA). Birds were maintained according to the guidelines set by this Animal Ethics Committee. All experimental protocols involving animals adhered to ARRIVE guidelines.

### Bacterial strains

*Salmonella enterica* subspecies *enterica* serovar Typhimurium (ST) strain 5591 and serovar Gallinarum strain E76 were procured from the National *Salmonella* Centre Repository, Indian Council of Agricultural Research-Indian Veterinary Research Institute (Izatnagar, India). Both strains were tested for their purity, morphology, and biochemical parameters. Isolated colonies of both serovars (*S*. Gallinarum, and *S*. Typhimurium) were grown on Hektoen Enteric Agar (HEA) plates. They were cultured overnight at 37 °C into freshly prepared Luria–Bertani (LB) medium. The overnight culture was transferred to a freshly prepared LB medium and cultured for an additional 2 h to achieve mid-log phase growth (optical density at 600 nm (OD_600_) = 0.5–0.6). The mid-log grown bacterial cultures were pelleted, washed with and suspended in RPMI-1640 media for subsequent use in various experiments described below.

### Isolation and culture of chicken blood monocyte-derived dendritic cells (chMoDCs)

White Leghorn broiler chicks (3 weeks old) were procured from the Indian Council of Agricultural Research-Central Avian Research Institute Hatchery (Izatnagar). They were screened for the presence of *Salmonella* species by the previously described method^13^.

Chicken DCs were prepared from buffy coats obtained from whole blood (collected via wing veins) in sterile vacutainers containing heparin and diluted with an equal volume of phosphate-buffered saline (PBS). Peripheral blood mononuclear cells (PBMCs) were isolated using the density-gradient method (Histopaque-1077; Sigma Life Sciences, Saint Louis, MO, USA). The cell pellet was suspended by addition of 500 μl of prewarmed RPMI-1640 medium (Gibco Life Technologies, Carlsbad, CA, USA). The concentration and viability of cells were determined using Trypan Blue (0.4%) staining. In vitro culture of chMoDCs was done according to the method described earlier with slight modifications^14^. PBMCs (2 × 10^6^ cells/mL) were cultured in 24-well plates in RPMI-1640 complete medium containing 8% chicken serum, 2% fetal bovine serum (FBS), 1% non-essential amino acids, 1% l-glutamine, penicillin (1 U/mL) and streptomycin (1 μg/mL) at 37 °C in an atmosphere of 5% CO_2_ for 6 days using recombinant chicken granulocyte–macrophage colony stimulating factor (GM-CSF; 20–50 ng/mL) (Abcam, USA) and interleukin (IL)-4 (10–30 ng/mL) (Abcam, USA). On every second day, three-quarters of the medium was replaced with fresh, prewarmed complete RPMI-1640 medium containing GM-CSF and IL-4 to remove non-adherent cells. The growth and differentiation of cells was recorded by observing the morphology, cells aggregation, and growth pattern of cells every second day up to day-6 of culture. On day-6, chMoDCs were infected with *S*. Gallinarum and *S*. Typhimurium at a multiplicity of infection (MoI) of 10 for different intervals according to the needs of different assays. Cells treated with lipopolysaccharide (LPS; (1 μg/mL) from *Salmonella enterica* serovar Typhimurium (Sigma, USA) and mock-treated cells were used as positive and negative controls, respectively. Morphologic changes in chMoDCs following infection with *S*. Gallinarum or *S*. Typhimurium were recorded ≤ 24 h post-infection.

### Intracellular survival assay (gentamicin protection assay)

An assay to measure intracellular bacterial survival was undertaken according to the previously described method with slight modifications^15^. Following infection of chMoDCs with respective *Salmonella* serovars for 1.5 h, the medium was replaced with fresh RPMI-1640 containing gentamicin (50 μg/mL). Cells were incubated further at 37 °C in an atmosphere of 5% CO_2_ for an additional 1.5 h to allow killing of extracellular bacteria. At 3 h after infection, cell lysates were prepared by addition of 0.1% Triton X-100 for bacterial counting, or the medium was replaced with fresh RPMI-1640 containing gentamicin (25 μg/mL) and re-incubation undertaken. Cell lysates were prepared at 24 h and 48 h after infection as described above, and counting of viable bacteria was done by plating serial dilutions of lysates on LB agar medium. Bacterial counts for respective intervals (3, 24 and 48 h) were determined and expressed as CFU/mL.

### Determination of toxicity to chMoDCs using the lactate dehydrogenase (LDH) assay

The toxic potential of both *Salmonella* serovars on chMoDCs was evaluated according to the method described by Rayamajhi and coworkers with slight modifications^16^. The toxicity mediated by *S*. Gallinarum and *S*. Typhimurium against chMoDCs was measured using the CytoTox 96^®^ Non-Radioactive Cytotoxicity Assay (Promega, Madison, WI, USA), which detects the stable cytosolic enzyme LDH released from lysed cells. Briefly, 6 day-cultured chMoDCs in 96-well round-bottom microtiter plates served as the target cells, which were infected with *S*. Gallinarum or *S*. Typhimurium for 3 h or 48 h with a MoI of 10. Fifty-microliter aliquots from all test wells and control wells were transferred to a fresh 96-well flat-bottom plate and mixed with 50 μl of CytoTox 96 Reagent. The plate was incubated for 30 min in the dark at room temperature. The reaction was stopped by addition of Stop Solution (50 μl) and OD recorded at 492 nm within 1 h.

### Complementary DNA (cDNA) synthesis and real-time quantitative polymerase chain reaction (RT-qPCR)

chMoDCs treated with *S*. Gallinarum, *S*. Typhimurium or LPS were harvested at 0 h (immature) as well as 6 h and 24 h after treatment. Total RNA was extracted using Ribozol™ RNA Extraction Reagent (Amresco, Solon, OH, USA) and subsequent precipitation in isopropanol using a standard protocol. cDNA synthesis was carried out in 20-μl volume using the RevertAid™ First Strand cDNA Synthesis Kit (Fermentas, Glen Burnie, MD, USA) according to manufacturer instructions. RT-qPCR was carried out using Quantifast™ SYBR^®^ Green PCR Master Mix (Qiagen, Hilden, Germany) with a RT-qPCR instrument (CFX96 Touch™; Bio-Rad Laboratories, Hercules, CA, USA). The fold-change in mRNA expression of the surface markers/costimulatory molecules of chMoDC (cluster of differentiation (CD)40, CD80, CD83, CD86, Major Histocompatibility Complex (MHC)-II), cytokines (tumor necrosis factor (TNF)-α, IL-12p35, interferon (IFN)-γ etc.), chemokines (C-X-C motif ligand 1 (CXCLi)1, CXCLi2) and toll-like receptor (TLR)-4 and TLR-21) from bacteria-infected and control groups was quantified using published primers (Supplementary Table S1).

Beta-actin was employed as an endogenous reference gene to calculate ΔCt values for each target gene. Previously, this has been established that β-actin is a stable endogenous reference gene for RT-qPCR studies^17^. The individual sample was run in triplicate, each with a 20-μl reaction. Briefly, 10 ng of cDNA (1 μl) was mixed with 0.2 μl of each forward and reverse primers (10-pmol each) with 10 μl of 2 × SYBR Green Master Mix in a final volume of 20 μl with the following cycling conditions: one initial cycle at 95 °C for 5 min followed by 40 cycles of amplification with denaturation at 95 °C for 10 s, annealing at 47–64 °C for 30 s (for different genes) and extension at 72 °C for 30 s. The specificity of primers was confirmed by the amplification plot and dissociation curve. The 2^−ΔΔct^ method was employed to ascertain the relative expression of each target gene in *Salmonella*-infected chMoDcs as the fold-difference from the mock-infected control group (immature chMoDCs)^18^. The LPS-treated group (positive control) was used to access chMoDC maturation.

### Mixed lymphocyte reaction (MLR) assay

The MLR assay was undertaken according to the method described by Cheminay and coworkers with slight modifications^19^. All reactions were set up in triplicate. Briefly, chMoDCs were cultured up to 6 days in 96-well plates as described above. On day-6, chMoDCs were infected with *S*. Gallinarum or *S*. Typhimurium at MoI = 10 in triplicate wells along with respective controls. After 1 h, non-internalized bacteria were removed by two washes with PBS. To kill the remaining extracellular bacteria, infected chMoDCs were incubated in medium containing gentamicin (100 μg/mL) for 1 h. After washing, chMoDCs were incubated further in the presence of medium containing gentamicin (25 μg/mL) for 24 h. The absence of extracellular bacteria was tested by plating supernatants onto HEA plates.

To carry out the MLR assay, T-lymphocytes were isolated and purified from allogenic chicken spleens (from chickens aged 3–6 weeks) using a nylon-wool column^20^. Purified T-cells were added to *S*. Gallinarum- or *S*. Typhimurium-primed chMoDCs (10:1) and incubated at 37 °C in an atmosphere of 5% CO_2_ for 72 h. The tetrazolium dye 3-(4,5-dimethylthiazol-2-yl)-2,5-diphenyltetrazolium bromide (MTT) was added to each well (20 μL/well) and the plate incubated further at 37 °C in an atmosphere of 5% CO_2_ for 4 h. Finally, dimethyl sulfoxide was added (50 μL/well) to dissolve formazon crystals and the OD was measured using an enzyme-linked immunosorbent assay plate reader at 570 nm to determine the Stimulation Index (SI).

### Statistical analyses

Statistical analyses were carried out with JMP (www.jmp.com) using the analysis of variance (ANOVA) and Student’s *t*-test. Data were analyzed using two-way repeat measures ANOVA followed by Bonferroni post-hoc test (when there were more than two time points) to detect differences between treatment groups. Differences were considered significant where *P* < 0.05. The correlation plot was prepared using SPSS 16.0. Bar charts were prepared using Prism 8.0 (GraphPad, San Diego, CA, USA).

## Results

### Characterization of chMoDCs

**Figure 1:**
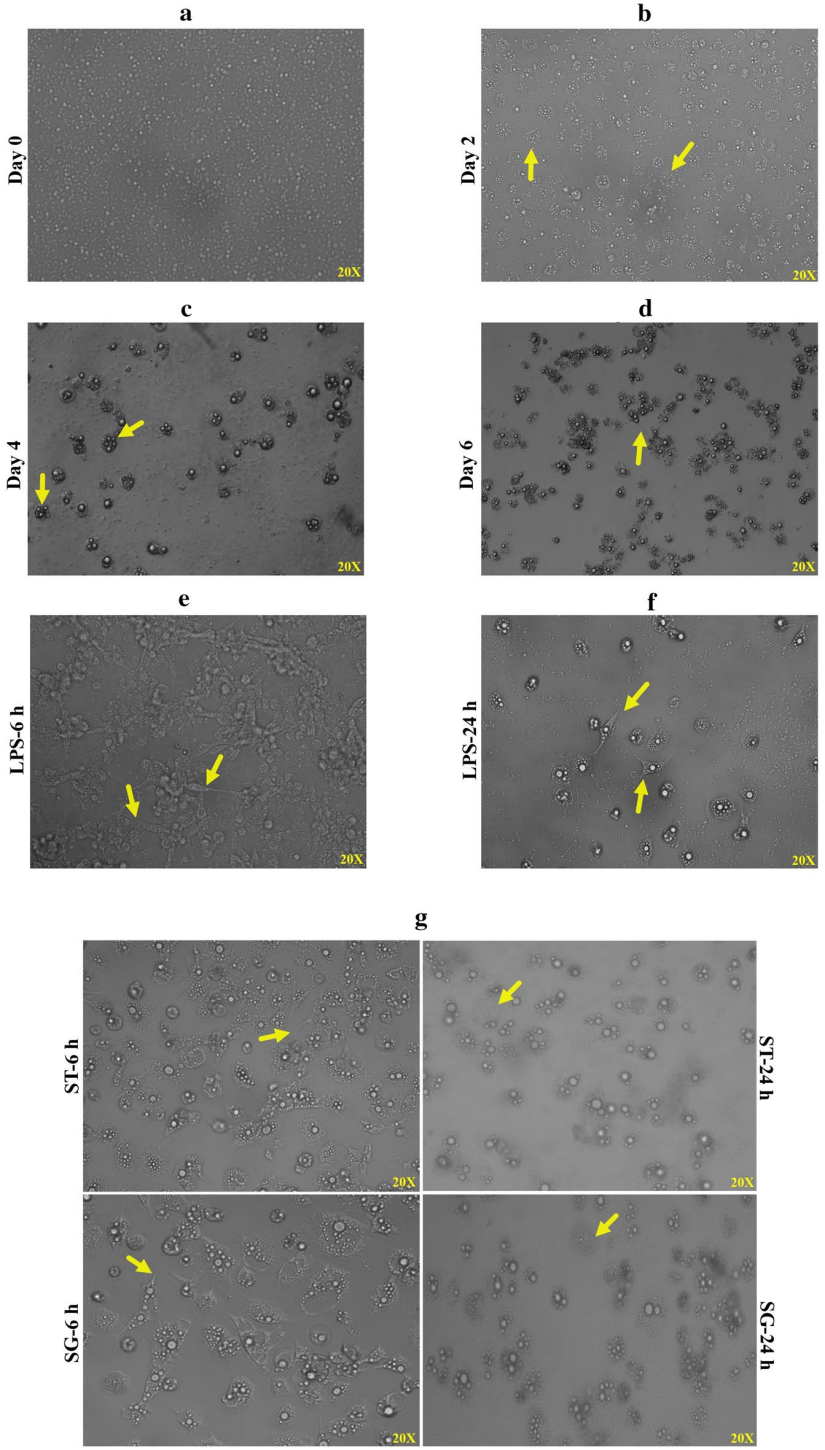
Morphology of chicken blood monocyte derived dendritic cells (chMoDCs) observed directly from cell culture plates using an inverted light microscope at ×200 magnification. Peripheral blood mononuclear cells (PBMCs) culture supplemented with recombinant chicken GM-CSF at 25 ng/mL and IL-4 at 12.5 ng/mL concentration followed by induction of maturation on 6th day using *Salmonella* LPS (1 µg/mL) for 24 h. (**a**) PBMCs were plated at a concentration of 2 × 10^6^ cells/mL. (**b**) Cell aggregates started to form. (**c**) Individual cell aggregates were distinctly evident. (**d**) Cells were firmly adhered with a network of dendrites started to form. (**e**) After 6 h of LPS stimulation, formation of extensive network of dendrites resembling typical cell morphology was evident. (**f**) After 24 h of LPS stimulation, drastic reduction in dendrite sizes were apparent. (**g**) chMoDCs infected with *S*. Gallinarum and *S*. Typhimurium at a multiplicity of infection of 10 showed typical cell morphology with extensive dendrites at 6 h post-infection, however during late phase of infection (24 h), cellular damage was evident as cells were found to be missing from within the cell aggregates with complete loss of dendrites.

**Figure 2:**
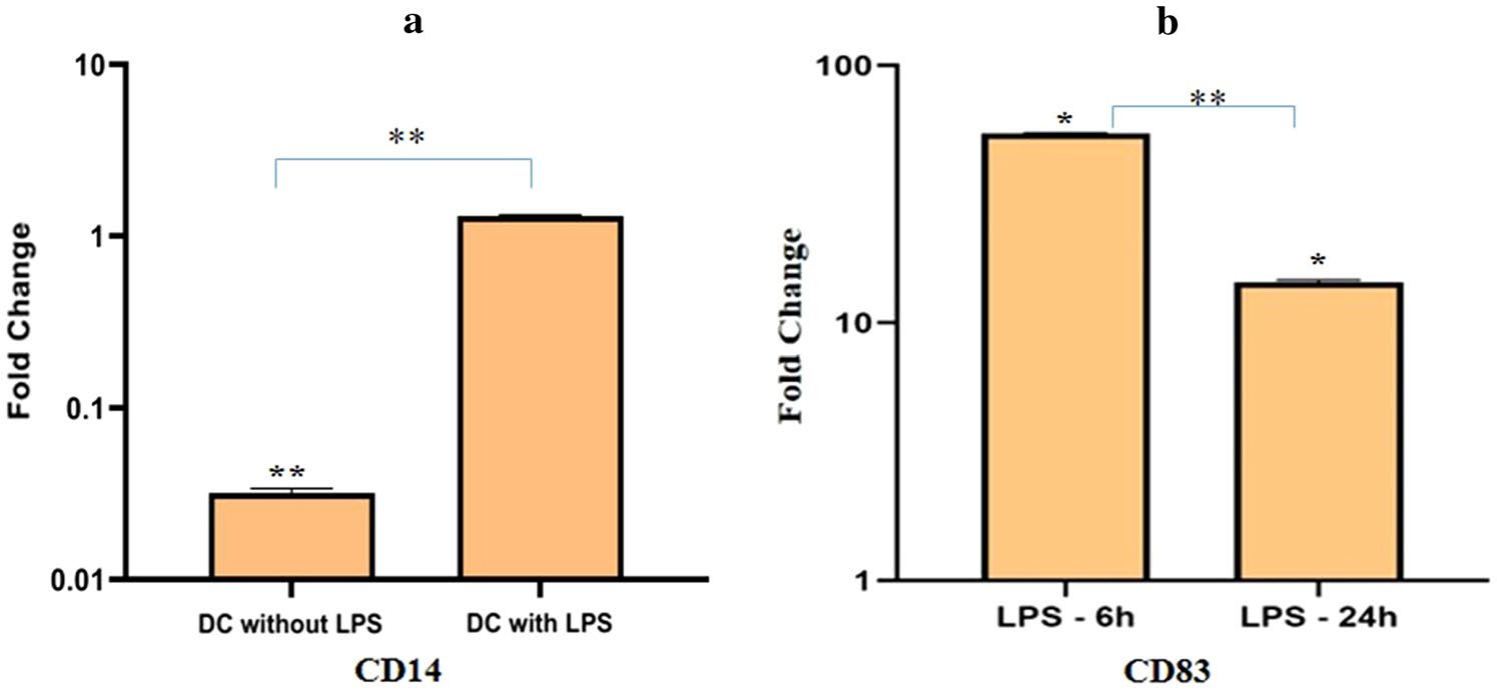
Fold change expression of cell surface molecules (CD14 and CD83) on chMoDCs 0 h and 24 h after exposure to *Salmonella* LPS (1 µg/mL). The mRNA expression for CD14 and CD83 was determined by real time-quantitative polymerase chain reaction (RT-qPCR); (**a**) data were analysed by Tukey’s multiple comparisons test to detect differences in mRNA expression of CD14 on immature and mature chMoDCs in relation to PBMCs. Values shown are mean ± SEM of three independent experiments (**P < 0.0001); (**b**) the fold-change expression of CD83 on chMoDCs at 6 h and 24 h post-LPS treatment in relation to immature DCs. Data were analysed by *t*-test. Values shown are mean ± SEM of three independent experiments (**P < 0.0001).

Cultured cells were characterized as chMoDCs based on their morphologic changes on alternate days and mRNA expression of CD14 and CD83 (maturation marker). Cell aggregation due to proliferation and stimulation of mononuclear cells by cytokines increased from day-2 to day-4 (Fig. 1a–c). These aggregates sustained the growth and differentiation of mononuclear cells, exhibiting a veiled or dendritic appearance that was most pronounced on day-6 (Fig. 1d). When PBMCs were cultured under the similar conditions as above without adding GM-CSF and IL-4, cells exhibited no aggregation and dendritic appearance on respective days of culture. Following treatment with LPS or both *Salmonella* serovars, most cells exhibited a characteristic structure with extensive dendrites at 6 h post-treatment (Fig. 1e–g). This finding suggested that chMoDCs were in the final stages of maturation. Expression of CD14 mRNA on immature and LPS-treated mature chMoDCs was compared with that obtained against unstimulated PBMCs. Immature chMoDCs showed relative downregulation (0.032 ± 0.0.002-fold), but CD14 expression was upregulated slightly in mature chMoDCs in comparison with that of unstimulated PBMCs (1.3 ± 0.02-fold; n = 3) (Fig. 2a). Besides, CD83 expression was upregulated on chMoDCs at 6 h (56-fold) and 24 h (16-fold) after LPS induction in comparison with that for immature phenotypes (*P* < 0.05) (Fig. 2b).

### Intracellular bacterial survival in chMoDCs

**Figure 3:**
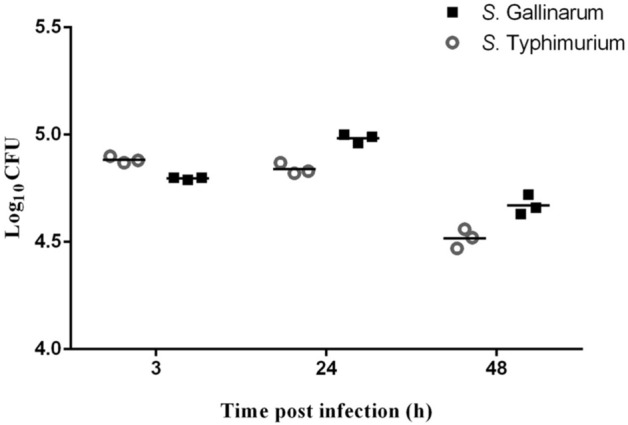
Intracellular survival of *S*. Gallinarum and *S*. Typhimurium in chicken dendritic cells (chMoDCs). The chMoDCs were treated with *S*. Gallinarum and *S*. Typhimurium for 90 min with a multiplicity of infection of 10:1 and viable bacterial counting was done on HEA plate following lysis of cells at 3, 24 and 48 h post-infection. Individual data point represents an independent experiment with the horizontal bars showing the corresponding arithmetic mean.

Gentamicin protection assay was employed to determine the intracellular bacterial survival in chMoDCs*.* The viable counts of *S*. Typhimurium recovered from chMoDCs was significantly higher than *S*. Gallinarum at 3 h post-infection (Fig. 3). *Salmonella*-infected chMoDCs showed a marginal increase in the viable counts of *S*. Gallinarum over a period from 3 to 24 h post-infection (*P* < 0.0001), however, *S*. Typhimurium remained almost constant or slightly decreased during this period (Fig. 3). This finding suggested that *S*. Gallinarum was multiplying in chMoDCs and was able to resist killing. At 48 h post-infection, the viable bacterial counts of both *Salmonella* serovars declined appreciably than that at 3 h post-infection, but *S*. Gallinarum continued to show higher persistence than that of *S*. Typhimurium (*P* < 0.0001). Hence, *S*. Gallinarum was able to resist killing by chMoDCs efficiently (Fig. 3 and Table 1).

**Table 1:** Intracellular survival and cytotoxicity % of *S*. Gallinarum and *S*. Typhimurium in the chicken monocyte derived dendritic cells (chMoDCs).

Strain	Intracellular survival log10 CFU (mean ± SEM)	% Cytotoxicity (mean ± SEM)
***S. Typhimurium***		
3 h	4.88 ± 0.008^b^	2.37 ± 0.43^c^
24 h	4.84 ± 0.015^b,c^	–
48 h	4.52 ± 0.02^e^	35.3 ± 4.10^bc^
***S. Gallinarum***		
3 h	4.80 ± 0.003^c^	29.67 ± 9.11^b,c^
24 h	4.98 ± 0.01^a^	–
48 h	4.67 ± 0.02^d^	5.77 ± 2.55^c^

Different superscripts (a, b, c, d, e) across the rows denotes significant difference.

### Cytotoxicity

**Figure 4:**
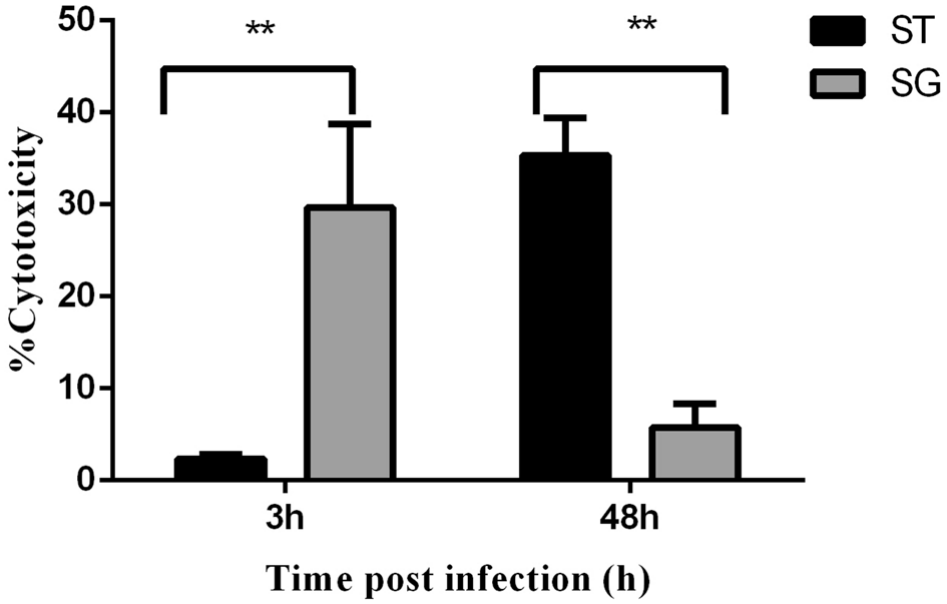
Measurement of cytotoxicity in chMoDCs induced by *S*. Gallinarum (SG) and *S*. Typhimurium (ST) with a multiplicity of infection of 10. The cytotoxicity level was determined by measuring lactate dehydrogenase (LDH) released to the cell culture supernatant at 3 and 48 h post-infection. Data were analysed by two-way repeat measures ANOVA for *S.* Typhimurium and *S.* Gallinarum at 3 and 48 h post-infection. Values shown are mean ± SEM of triplicates from three independent experiments and expressed as percentage relative to lysis buffer treated cells (mean) (**p < 0.001).

The viability of chMoDCs in response to *Salmonella* infection was determined by measuring the LDH level in cell culture supernatants. Results were expressed as the percentage of LDH released by infected cells in comparison with LDH released by lysis buffer-treated (lysed) monolayers at 3 h and 48 h post-infection. Nil or a very low level of cytotoxicity was recorded in the *S*. Typhimurium group as compared with that in the *S*. Gallinarum group, which induced a high level of toxicity in chMoDCs at 3 h post-infection (*P* < 0.001) (Fig. 4). However, the level of cytotoxicity in the *S*. Gallinarum group was decreased at 48 h post-infection and was below the level of *S*. Typhimurium (*P* < 0.001) (Fig. 4 and Table 1).

### Expression of costimulatory molecules on *Salmonella*-treated chMoDCs

**Figure 5:**
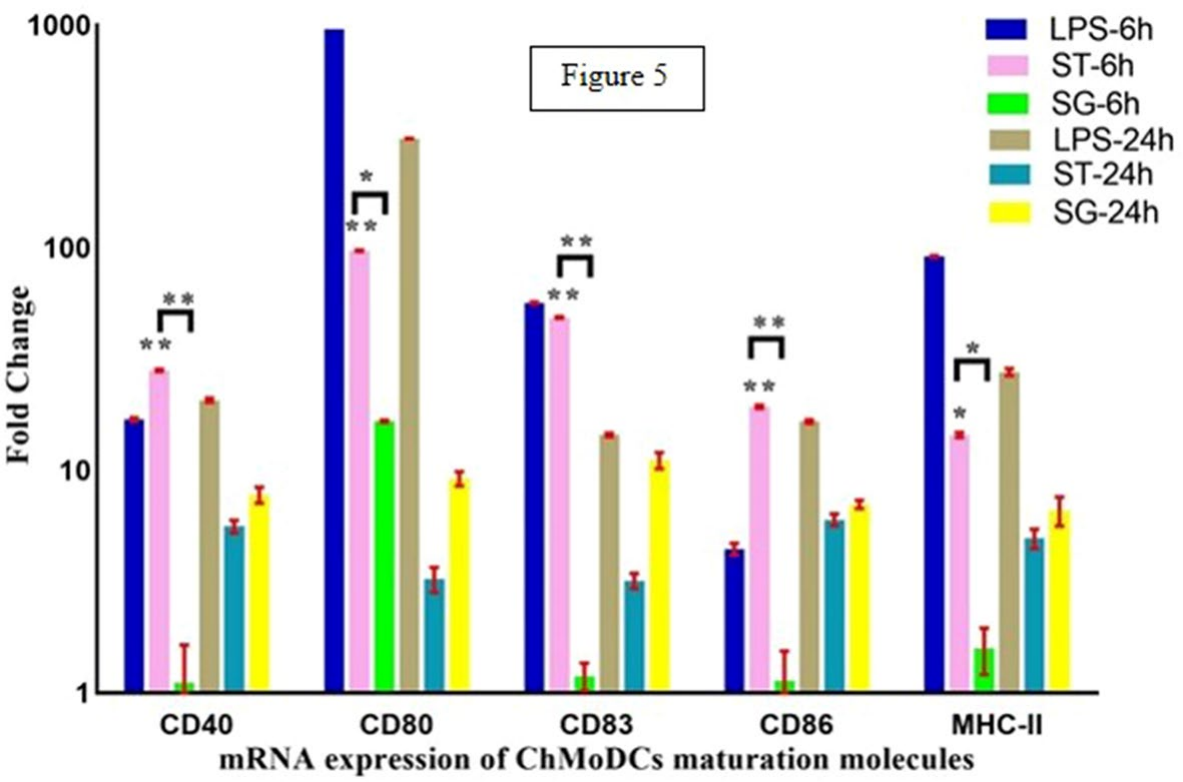
Fold change expression of cell surface molecules on chMoDCs 6 h and 24 h after exposure to *S*. Gallinarum (SG) and *S*. Typhimurium (ST) (multiplicity of infection was 10) and *Salmonella* LPS. The mRNA expression for CD40, CD80, CD83, CD86 and MHC-II was determined by real time-quantitative polymerase chain reaction (RT-qPCR). The mock-infected cells were used as control for calculating the fold change of differential gene expressions in the infected cells. Data were analysed by two-way repeat measures ANOVA for *S.* Typhimurium and *S.* Gallinarum at 6 and 24 h post-infection. LPS served as positive control but not included in the analysis. Data for negative control is not shown. Values shown are mean ± SEM of three independent experiments (*P < 0.05, **P < 0.001).

The fold-change in mRNA expression of several molecules involved in antigen presentation and stimulation of T-cells in chMoDCs at 6 h and 24 h post-infection by *S*. Gallinarum and *S*. Typhimurium was evaluated by RT-qPCR. Expression of all surface molecules was upregulated in the *S*. Typhimurium group at 6 h post-infection (*P* < 0.001). It showed a reduction during the late phase of infection (24 h) but remained significantly higher than that of mock-infected controls (three- to six-fold) (Fig. 5). *S.* Gallinarum induced relatively delayed upregulation of expression of all surface molecules except CD80, and the effect was maximal at 24 h (*P* < 0.001). Expression of CD80 mRNA was numerically greater among all other surface molecules in both groups at 6 h post-infection (Fig. 5 and Supplementary Table S2).

### TLR expression in response to *Salmonella* infection

**Figure 6:**
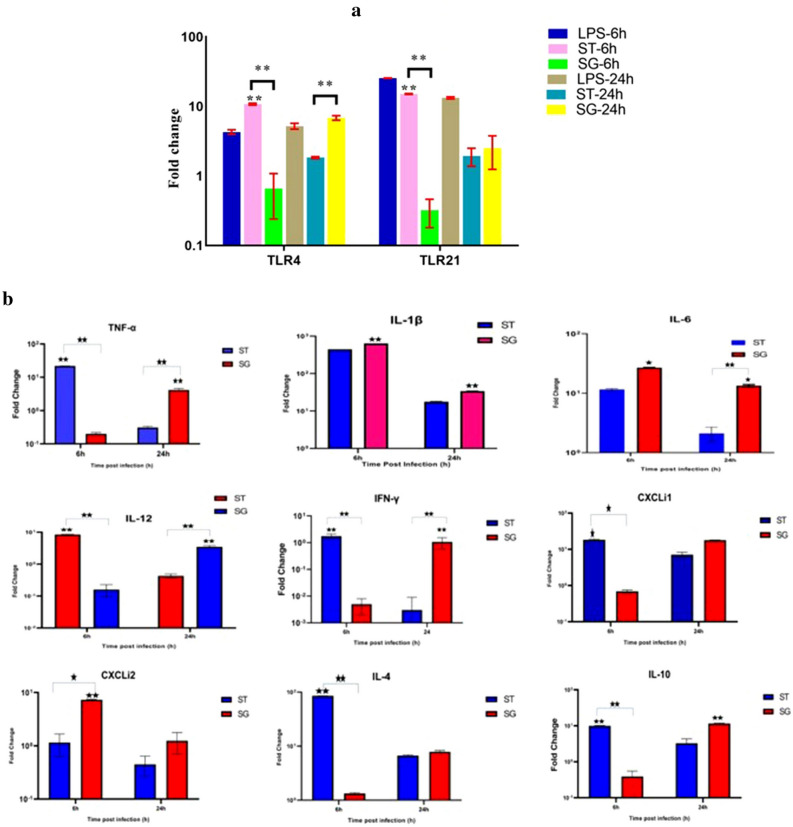
Expression of TLRs (**a**) and cytokines (**b**) in chMoDCs at 6 and 24 h post-infection with *S*. Gallinarum (SG) and *S*. Typhimurium (ST) (multiplicity of infection 10). The mRNA expression for pro- and anti-inflammatory cytokines, related key chemokines, and toll like receptors were determined by real time-quantitative polymerase chain reaction (RT-qPCR). The mock-infected cells were used as control for calculating the fold change of differential gene expressions in the infected cells. (**a**) Data were analysed by two-way repeat measures ANOVA for *S.* Typhimurium and *S.* Gallinarum at 6 and 24 h post-infection. LPS served as positive control but not included in the analysis. Data for negative control is not shown; (**b**) Data were analysed by two-way repeat measures ANOVA for *S.* Typhimurium and *S.* Gallinarum at 6 and 24 h post-infection. Data for negative control served as calibrator group to calculate the fold change differences. Values shown are mean ± SEM of three independent experiments (*P < 0.05, **P < 0.001).

Similar to costimulatory and other surface expressed molecules, mRNA expression of TLR4 (10.7-fold) and TLR21 (15-fold) was upregulated in *S*. Typhimurium-treated chMoDCs at 6 h post-infection (*P* < 0.001) but reduced almost to a basal level at 24 h post-infection (Fig. 6a). mRNA expression of both TLRs in the *S*. Gallinarum group was downregulated at 6 h post-infection, but this showed a peak at 24 h post-infection with fold-change being greater for TLR4 (6.82-fold) than for TLR21 (2.5-fold) (Fig. 6a and Supplementary Table S3).

### Expression of cytokine and chemokine genes in response to *Salmonella* infection

The fold expression of proinflammatory cytokines (IL-1β, IL-6) was upregulated significantly in the *S*. Gallinarum group and *S*. Typhimurium group at all time points in comparison with that in the mock-infected control (Fig. 6b). However, a declining trend in their fold expression was observed during the late phase of infection (24 h post-infection) in both groups. Upregulation of TNF-α expression was observed only at 6 h and 24 h post-infection in the *S*. Typhimurium (21.8-fold) group and *S*. Gallinarum (4.18-fold) group, respectively (Fig. 6b). mRNA expression of IL-1β was greatest among all other cytokines and chemokines at all time points in both groups, with the fold-change being greater in the *S*. Gallinarum group than that in the *S*. Typhimurium group (Fig. 6b). Neither *Salmonella* serovars showed a significant upregulation in IFN-γ mRNA expression as compared with that of mock-infected controls at any time point (Fig. 6b). Upregulation of IL-12p35 expression was observed in the *S*. Typhimurium group (8.45-fold) at 6 h post-infection, whereas *S*. Gallinarum and *S*. Typhimurium groups showed downregulation of IL-12 expression at 6 h and 24 h post-infection, respectively (Fig. 6b). Upregulation of CXCLi1 expression was observed in both groups at all-time points except in the *S*. Gallinarum group at 6 h post-infection, whereas CXCLi2 expression was upregulated only at 6 h in the *S*. Gallinarum group (Fig. 6b). *S.* Typhimurium treated chMoDCs showed upregulation of expression of IL-4 and IL-10 mRNA at all time points. However, in the *S*. Gallinarum group, downregulation of expression of both the cytokines (IL-4 and IL-10) mRNA was evident at 6 h post-infection in comparison to mock-treated cells (Fig. 6b and Supplementary Table S4).

### Stimulation of allogenic T-cells by *Salmonella* treated chMoDCs (MLR assay)

**Figure 7:**
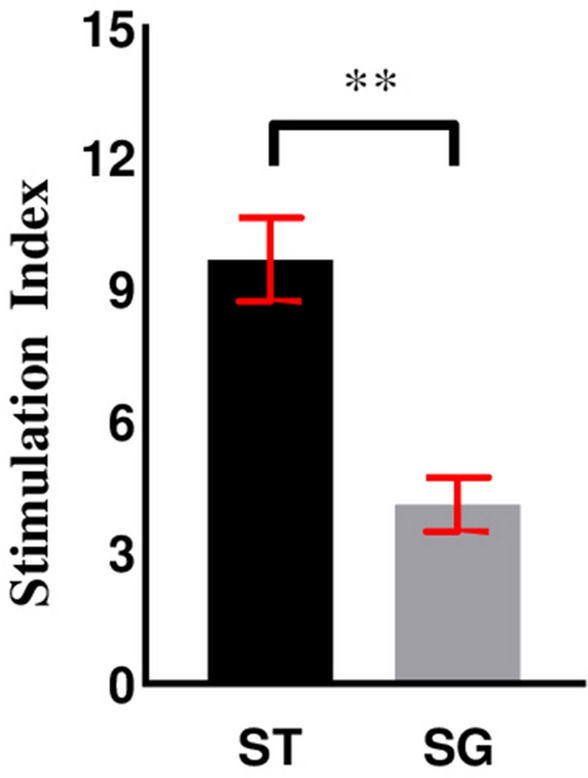
Stimulatory capacity of chMoDCs to allogenic chicken T-cells following infection with *S*. Gallinarum (SG) and *S*. Typhimurium (ST). chMoDCs were harvested 24 h following exposure with *S*. Gallinarum and *S*. Typhimurium with a multiplicity of infection of 10 and cells were washed and processed for mixed lymphocyte reaction (MLR). Allogeneic chicken T-cells were incubated in triplicates with chMoDCs (10:1) for 72 h. Data were analysed by independent *t*-test. Results are expressed as mean stimulation index (SI) ± SD and data are representative of two independent experiments (**P < 0.001).

The function of chMoDCs exposed to *S*. Gallinarum or *S*. Typhimurium to stimulate proliferation of allogenic T-cells was tested and compared with that of mock-infected chMoDCs and between treatment groups. *S*. Typhimurium-exposed chMoDCs had a higher stimulatory potential towards allogenic T-cells (SI = 9.7) in comparison with that of *S*. Gallinarum infection (SI = 4.1) (*P* < 0.001) (Fig. 7).

## Discussion

Mechanisms of host adaptation can differ between host-restricted and broad-host-range (host-generalist) strains of *Salmonella* serovars. Reports have suggested that *Salmonella* serovars differ in their ability to avoid adaptive immunity in mice because of interference with DC function, and that this interference feature of *Salmonella* is host-restricted^21^. To what extent *S.* Gallinarum modulates the function of chicken DCs (and whether this feature confers host specificity to this serovar) is not known. Therefore, we investigated in detail the interplay between host-restricted *S.* Gallinarum and host-generalist *S.* Typhimurium with chicken DCs.

DCs derived from chicken bone marrow or blood monocytes have been cultured and characterized by several research groups^17,22,23^. We have used PBMCs as a source of monocytes and optimized the concentration of GM-CSF (25 ng/mL) and IL-4 (12.5 ng/mL) for the proper differentiation of monocytes into chMoDCs. In the present study, chMoDCs were grown and characterized according to their typical morphology and fold-change in mRNA expression of CD14 and CD83. Most cells exhibited a typical dendrite-like structure which was visible microscopically (Fig. 1d). In general, T-cells, DCs, and platelets are CD14-negative cells, but bone marrow-derived DCs can express CD14 to various extents during differentiation^24^. We observed a significant downregulation of CD14 mRNA expression in immature chMoDCs on day-6 of culture as compared with that of blood monocytes/PBMCs (which were originally taken on day-0 for in vitro culture of chMoDCs) (*P* < 0.0001). However, following treatment of chMoDCs with bacterial LPS at 24 h of incubation, we documented a slight increase in the fold-change expression of CD14 as compared with that of blood monocytes/PBMCs (1.3 ± 0.02-fold) (Fig. 2a). This observation is in accordance with earlier report showing surface expression of CD14 to be upregulated positively with incubation of mouse bone marrow-derived DCs with LPS^25^. Therefore, CD14 could show marginal expression on mature chicken DCs. Reports describing CD14 expression on immature or mature phenotypes of chMoDcs have not yet been published. CD14 expression was observed on monocytes and mature chMoDCs, so it cannot be considered to be a specific marker for DC characterization. Another surface marker, CD83, which is invariably considered to be a maturation marker for DCs, has been reported in mammals and avian species^23,26^. The fold-change expression of CD83 on chMoDCs was upregulated significantly at 6 h and 24 h post-LPS treatment as compared with that in untreated cells (*P* < 0.05) (Fig. 2b). Besides LPS, both serovars of *Salmonella* favored maturation of chMoDCs in comparison with mock-infected cells. However, fold-change expression of CD83 mRNA (maturation level) was significantly greater in *S.* Typhimurium-infected chMoDC as compared with that in the *S*. Gallinarum group (Fig. 5 and Supplementary Table S2). This finding suggested the potential of the host-restricted serovar *S*. Gallinarum for delaying the DC maturation.

Both *Salmonella* serovars imparted pathologic/morphological changes in the chMoDCs in comparison with that in the mock-treated control. The chMoDCs treated with both the serovars got damaged as cells were found to be missing from within the cell aggregates with complete loss of dendrites which were evident at early stage of infection (6 h). These changes were more pronounced at 24 h post-infection (Fig. 1g). Use of a high ratio of *Salmonella*:DCs (10:1) might be the reason behind these pathologic changes because there have been reports of a reduction in the viability of murine DCs ≤ 50% upon infection with a higher number (10:1) of *Salmonella* per cell^27^. However, other scholars have reported no toxicity towards DCs even when 15 bacteria (*S.* Typhimurium) per cell were used^28^. A high ratio of bacteria:DCs is unlikely to equate to high ratios in vivo in the early stage of infection, but may be more relevant to the later stage of infection, when marked pathologic changes become apparent. Hence, a low-dose model of infection might be more relevant for studies on host–pathogen interactions because this may have a close resemblance to in vivo situations. This hypothesis warrants further studies using a low-dose model of infection.

The ability to survive in the intracellular environment is central to the pathogenesis of *Salmonella* infection. Our findings suggest that chMoDCs supported the replication of *S*. Gallinarum, which resulted in a marginal increase in their numbers over a period from 3 to 24 h post-infection (*P* < 0.0001), though the initial viable counts at 3 h post-infection was significantly higher for *S.* Typhimurium (Fig. 3 and Table 1). Recovery of high viable counts of *S.* Typhimurium at 3 h post-infection may be due to its relatively higher invasive potential and low cytotoxicity, in agreement with earlier publication^29^. The survival of *S*. Gallinarum at 24 h and 48 h post-infection was higher than that of *S.* Typhimurium (*P* < 0.0001), which suggested that, between the two serovars, *S*. Gallinarum was more resistant to intracellular killing by chMoDCs (Fig. 3 and Table 1). Our data show that *Salmonella* could persist in infected chMoDCs for ≥ 48 h post-infection, though the survival of bacteria declined over time. This could be one of the factors responsible for the higher persistence and disease-causing ability of *S*. Gallinarum in chickens as compared with that of *S.* Typhimurium because the survival for *S*. Gallinarum was notably higher in chMoDCs than that of *S.* Typhimurium. This finding is contrary to reports showing host-restricted and host-generalist *Salmonella* serovars do not exhibit a marked difference in their resistance to killing of chicken macrophages at 48 h of infection^15^. The reason for this discrepancy might be due to the use of different phagocytic cells (DCs vs. macrophages), which could modulate the replication of bacteria differently.

*Salmonella* species have been shown to promote the death of host cells as early as 1 h post-infection in a SPI-2 T3SS-dependent manner^30,31^. *S.* Gallinarum-infected chMoDCs showed a significantly higher level of cytotoxicity (29.67 ± 9.11) at 3 h post-infection in comparison with that of *S.* Typhimurium (2.37 ± 4.03), which was relatively more cytotoxic at the late phase of infection (48 h) (Fig. 4 and Table 1). Our results are in accordance with a previous report which revealed that host-restricted *Salmonella* serovars such as *S.* Typhimurium induced rapid cytotoxicity in mouse bone marrow derived DCs at 3 h post-infection^32^. This could probably dampens the adaptive immunity against this gut pathogen and has been proposed as an early immune escape mechanism for subsequent systemic spread. Hence, we propose that the capacity of *S*. Gallinarum to restrict the activation of chMoDCs during early stage of infection may be linked with its higher cytotoxic potential at 3 h post-infection.

We measured cytotoxicity in the early phase (3 h) and late phase of infection (48 h). By doing so, it was possible to distinguish between strains that were recovered in low counts due to cytotoxicity and lysis of chMoDCs and those that succumbed to DC defenses. Hence, our cytotoxicity results were subjected to correlation with the intracellular bacterial survival assay which revealed no correlation between these assays for both the serovars over a period of time except for *S.* Gallinarum. The correlation plot revealed a significant positive correlation between these two assays at 48 h post-infection for *S.* Gallinarum (*P* < 0.05) (see Supplementary Fig. S1). A clear link to the superior survival of *S*. Gallinarum in chMoDCs was not evident by present data on intracellular survival assay because, after 48 h of infection, the level of cytotoxicity was very low for *S*. Gallinarum (5.77 ± 2.55%) as compared to *S.* Typhimurium (35.3 ± 4.10%) but bacterial recovery was only marginally higher for former than later serovar, in agreement with previous report^15^ (Figs. 3 and 4, Table 1). However, data for reduced cytotoxicity by *S*. Gallinarum at 48 h post-infection may not be correct in true sense as the replacement of media at 3 h post-infection witnessed loss of accumulated LDH and consequently low cytotoxicity. Indeed, the cumulative percentage cytotoxicity seem to be almost equal for both the serovars at 48 h post-infection. Nevertheless, further detailed studies on the interaction of chicken DCs with *Salmonella* are warranted to elucidate undefined host-specific traits.

DC maturation involves a coordinated series of events. An important hallmark of DC maturation is increased surface expression of MHC molecules and costimulatory molecules^33^. Expression of costimulatory molecules and MHC molecules was upregulated following treatment of chMoDCs with *Salmonella* serovars, findings that are consistent with those in other reports^28,34^._._ *S.* Typhimurium-infected chMoDCs showed significant upregulation in the expression of costimulatory (CD40, CD80, CD83 and CD86) and MHC molecules at 6 h post-infection in comparison to *S.* Gallinarum (Fig. 5 and Supplementary Table S2). This proves that later *Salmonella* serovar had retarded the maturation of DCs especially during the early phase of infection (6 h). This is the first report describing the differential expression of costimulatory molecules and MHC molecules on chicken DCs in response to host-restricted and host-generalist *Salmonella* serovars.

*Salmonella* possesses a range of protein and non-protein structures that function as pathogen-associated molecular patterns, which are recognized by pattern recognition receptors such as TLRs expressed on DCs. TLR4 and TLR21 (which is functionally equivalent to mammalian TLR9) recognize and bind with their respective ligands LPS and Unmethylated (or 5′—C—phosphate—G—3′) DNA sequences are considered to be key players in immunity against *Salmonella* infection. We recorded a significant upregulation in expression of TLR4 and TLR21 in *S.* Typhimurium-infected chMoDCs in comparison with that in the *S*. Gallinarum group during the early phase of infection (6 h) (Fig. 6a and Supplementary Table S3). This optimal stimulation of the innate immune response might be a contributing factor in the early clearance of *S.* Typhimurium, and poor induction of TLR expression by *S*. Gallinarum (especially during the early phase of infection) may be beneficial in establishing systemic infection by avoiding the initial innate immune response. Our observations stated above are contrary to those in a recent report of significant downregulation of expression of TLR2, TLR4 and TLR5 in *S.* Typhimurium- and *S.* Dublin-but not *S*. Gallinarum-infected HD11 cells at 6 h post-infection^35^. We hypothesize that use of opsonized bacteria at a low MoI (5) and different phagocytic cells might be the reason for this discrepancy because opsonized bacteria may not replicate efficiently inside cells and thereby lead to low induction of TLRs.

One of the most striking characteristics of DCs is their ability to produce cytokines and chemokines, which have important roles in regulating host immune responses upon *Salmonella* infection^36^. Hence, we evaluated the ability of chMoDCs to induce the transcription of several cytokines and chemokines in response to infection by *S*. Gallinarum or *S.* Typhimurium.

We found upregulation in expression of the proinflammatory cytokines IL-1β, IL-6, TNF-α, and IL-12p35 as well as the chemokine CXCLi1 in chMoDCs treated with *S.* Typhimurium during the early phase of infection (6 h). However, mRNA expression of only IL-1β, IL-6 and CXCLi2 was upregulated in the early phase (6 h) whereas mRNA expression of TNF-α, IL-12p35 and CXCLi1 was upregulated in the late phase (24 h) of *S*. Gallinarum infection (Fig. 6b and Supplementary Table S4). TNF-α is a crucial proinflammatory cytokine required during the early phase of infection and also during a specific immune response. TNF-α expression was downregulated during the initial phase (6 h) of *S*. Gallinarum infection. Switching to increased expression of TNF-α, IL-12p35 and CXCLi1 by *S*. Gallinarum-infected chMoDCs from 6 to 24 h post-infection might have been due to increased bacterial survival and higher expression of costimulatory molecules, which may have increased expression of these molecules synergistically during the late phase of infection (24 h). We concluded that *S.* Typhimurium triggered a strong inflammatory response which may limit the spread of bacteria largely to the gut. *S*. Gallinarum induced an inflammatory response that was not as strong as that induced by *S.* Typhimurium, especially during the early phase of infection. Hence, containment of infection was poor, and this could culminate in a severe systemic disease called fowl typhoid. Studies have revealed dominant proinflammatory-cytokine and chemokine responses by primary human and chicken epithelial cells as well as chicken macrophages in response to host-generalist serovar such as *S.* Typhimurium in comparison to host-restricted serovars^35,37,38^. Our findings are distinct in that *S*. Gallinarum did not completely retard the expression of proinflammatory cytokines as observed in epithelial cells^38^. However, their quantum was relatively narrower (IL-1β, IL-6 and CXCLi2) in comparison to *S*. Typhimurium (IL-1β, IL-6, TNF-α, IL-12p35 and CXCLi1) infected chMoDCs especially during early phase of infection (6 h)^29,35^. Induction of only a limited number of proinflammatory mediators by *S*. Gallinarum during the early phase of infection may correlate with its higher cytotoxic potential and poor capacity for DCs activation as revealed by low expression of co-stimulatory and MHC class II mRNA levels. There may be other contributing factors governing the differences in host responses against these serovars, including the difference in the early immune response.

There is a growing body of evidence suggesting that DCs can shape the T-helper type 1 (Th1)–Th2 balance, and that the latter is influenced primarily by the type of microbial interactions and their outcome^39^. This balance is governed by differential production of IL-12 and IL-4 by DCs because IL-12 induces IFN-γ-producing Th1 cells whereas Th2 responses are primed by IL-4^40^. We recorded significant upregulation of expression of IL-12p35, IL-4 and IL-10 and a slight increase in IFN-γ expression by *S.* Typhimurium-infected chMoDCs in comparison with that in the *S*. Gallinarum group at the early phase of infection (6 h), whereas *S*. Gallinarum favored strong upregulation of mRNA expression of IL-4, IL-10 and IL-12p35 (marginal increase) at the late phase of infection (24 h) (Fig. 6b and Supplementary Table S4). Hence, *S.* Typhimurium-treated DCs may favor polarization of naïve T-cells to Th1 cells and Th2 cells, which could facilitate early clearance from the host, whereas *S*. Gallinarum favored primarily the Th2 response during the late phase of infection. This diminished expression of Th1-polarizing cytokines by chMoDCs in response to *S*. Gallinarum points towards a suppressed immune response in the form of cell-mediated immunity against this intracellular pathogen. This action may be one of the ways by which *S*. Gallinarum modulates the defense of DCs, by interfering with their ability to stimulate naïve T-cells through production of Th1 cytokines and, thus, helping their survival within chMoDCs. This observation is consistent with a finding by Tang and colleagues indicating that, in comparison with a host-generalist *Salmonella* serovar (*S*. Enteritidis), a host-restricted serovar (*S*. Pullorum) failed to upregulate mRNA expression of IL-12 and IFN-γ in the spleen, which might have favored persistent infection in the spleen of infected chickens^41^.

It has been shown that *Salmonella*-exposed DCs favor the induction of allogeneic T-cell responses^42^. Co-culture of *Salmonella-*infected chMoDCs with allogenic chicken T-cells led to a significant increase in proliferation of lymphocytes in comparison with the levels shown by allogenic chicken T-lymphocytes in the presence of mock-infected DCs (Fig. 7). *S.* Typhimurium-treated chMoDCs had a higher potential for allogenic T-cell stimulation than that of *S*. Gallinarum (*P* < 0.001). These observations point to an enhanced capacity of chMoDCs to degrade *S.* Typhimurium, which can lead to efficient presentation of bacteria-expressed antigens on MHC class-I and class-II molecules. Hence, the capacity of *S*. Gallinarum to interfere with DC function could prevent activation of T-cells against antigens derived from this pathogen, a finding that is in concert with an observation by Bruno et al.^43^. Consistent with these findings, the impaired capacity of *S.* Typhimurium to survive within chMoDCs for an extended period could explain (at least in part) why adaptive immunity in chickens could be activated against this host-generalist pathogen. This observation is consistent with a report showing that *S.* Typhi and *S*. Enteritidis (non-host adapted serovars for mice) were degraded by murine DCs as compared with *S.* Typhimurium (host-adapted serovar for mice) but this condition was reversed by using human DCs within which *S.* Typhi (host-adapted serovar for humans) could replicate and *S.* Typhimurium and *S*. Enteritidis (non-host adapted serovars for humans) failed to replicate, and were degraded readily by these APCs^21^.

Taken together, our findings indicate that infection of chMoDCs with *S*. Gallinarum was characterized by low intracellular killing and delayed activation, possibly favoring long-term survival in the intracellular environment, and caused an overall low induction of proinflammatory responses along with a poor T-helper type-1 response. Overall, our results support a new component for the host specificity of *S*. Gallinarum: the capacity to interfere with DC function in chickens. This information could contribute to: (i) identification of new molecular factors determining the host specificity of *S*. Gallinarum; (ii) the design of new and improved vaccines against this intracellular pathogen.

## Supplementary Information

Supplementary Information.

## Data Availability

The authors declare that complete datasets generated for this study are included in the article/supplementary material. The datasets used or analyzed in present study are available through request to corresponding author.
