# Supplementary material for: Differential responses of chicken monocyte-derived dendritic cells infected with Salmonella Gallinarum and Salmonella Typhimurium
Source: Sci Rep. 2021 Aug 26;11:17214. doi: 10.1038/s41598-021-96527-w (PMC8390485; doi:10.1038/s41598-021-96527-w)

Differential responses of chicken monocyte-derived dendritic cells infected with *Salmonella* Gallinarum and *Salmonella* Typhimurium

Degpal Singh[a,1] Mithilesh Singh[a,*,1], Vishal Chander[2], Gaurav Kumar Sharma[2], Manish Mahawar[3], Aamir Salam Teeli[4], Tapas Kumar Goswami[1]

Supplementary Table 1. List of primers with their sequences and annealing temperature used for quantification of various immune response genes by real-time PCR

S. No.	Gene	Oligo Sequences	Annealing temp (^{0}C)	Accession No.
1	CD14	F- GGGGGGACCTGGAGAAATA<br>R- AGGAGAAGAACCGCAGGA	54	NM_001139478
2	CD40	F-AACGCAACGCACAACACTG<br>R-GTCCCTTTCACCTTCACCACA	57	EF554721.1
3	CD80	F-CAGCAAGCCGAACATAGAAAGA<br>R-AGCAAACTGGTGGACCTGAGAA	57	EF554723.1
4	CD83	F-GCCTACACTCTACTCTTCACCCTG<br>R-TATTCTGTCGCCAACTCC	47	XM_040663657.1
5	CD86	F-TGTAGGGATTG GGATGAGGG<br>R-CGCTGGAAGAGCAGGAAAGAT	57	EF554724.1
6	MHC IIα	F-GGGGTTTACGACAGCGTCTATT<br>R-TTCCGGGTCCCACATCCT	55	AY357256.1
7	TLR4	F-CCATCCACTCAGACAACCTTTCGA<br>R-AGTAAACGCAGCAGCACGCG	60	AY064697
8	TLR 21	F- GCAGCTCAGCCGCTCTTTT<br>R-CCTTCTTCTTCCTCCTCCTCT CC	55	NM_001030558.1
9	TNF-α	F-AGCTCTTTTGTG CCTGTTTATTTC<br>R-AAGCTTCAGTATGGGTTTAGAGA	53	NM_204267.1
10	IL-1β	F-AACATCGCCACCTACAAG<br>R-GACGGTAATGAAACATAAACG	54	AJ245728
11	Il-6	F-GCTCGCCGGCTTCGA<br>R-GTAGGTCTGAAAGGCGAACAG	54	AJ309540.1
12	IL- 12α	F-TGGCCGCTGCAAACG<br>R-ACCTCTTCAAGGGTGCACTCA	51	NM_213588.1
13	IFN-ʏ	F-AAGTCAAAGCCGCACATCAAACC<br>R-TGGATTCTCAAGTCGTTCATCG	54	DQ906156
14	IL-4	F-AACATGCGTCAGCTCCTGAAT<br>R-CTGCTAGGAACTTCTCCATTGAA	60	AJ621249.1
15	IL-10	F-CATGCTGCTGGGCCTGAA<br>R-CGTCTCCTTGATCTGCTTGATG	59	AJ621254.1
16	CXCLi 1	F-CCAGTGCATAGAGACTCATTCCAAA<br>R-TGCCATCTTTCAGAGTAGCTATGACT	57	NM_205018.1
17	CXCLi 2	F-GCCCTCCTCCTGGTTTC<br>R-TGGCACCGCAGCTCATT	54	NM_205498.1
18	Beta-actin	F-GCACCACACTTTCTACAATGAG<br>R-ACGACCAGAGGCATACAGG	59	NM_205518

Supplementary Table 2. Fold change expression of surface markers (Mean ± S.E.) following infection with *S*. Gallinarum and *S*. Typhimurium at 6 and 24 h post-infection. Different superscripts (a,b,c) across the columns denotes significant difference.

**Co-stimulatory molecules**	**ST-6 h**	**SG-6 h**	**ST-24 h**	**SG-24 h**
CD40	28.1±0.30 ^{c}	1.11±0.53 ^{a}	5.59±0.36^{b}	7.76±0.64^{bc}
CD80	97±0.68^{b}	16.6±0.19^{a}	3.24±0.42^{a}	9.18±0.68^{a}
CD83	48.6±0.39^{c}	1.19±0.17^{a}	3.19±0.25^{ab}	11.08±0.92^{b}
CD86	19.29±0.30^{c}	1.13±0.41^{a}	6.0±0.36^{b}	7.02±0.30^{bc}
MHC-II	14.4±0.36^{b}	1.58±0.37^{a}	4.94±0.49^{ab}	6.60±0.98^{ab}

ST-6 h & ST-24 h = S. Typhimurium at 6 h and 24 h post-infection; SG-6 h & SG-24 h = S. Gallinarum at 6 h and 24 h post-infection

Supplementary Table 3. Fold change expression of TLRs mRNA (Mean ± S.E.) following infection with *S*. Gallinarum and *S*. Typhimurium at 6 and 24 h post-infection. Different superscripts (a,b,c) across the columns denotes significant difference.

**TLRs**	**ST-6 h**	**SG-6 h**	**ST-24 h**	**SG-24 h**
TLR-4	10.77±0.33^{b}	0.66±0.42^{a}	1.83±0.06^{a}	6.82±0.50^{b}
TLR-21	15.1±0.19^{b}	0.32±0.14^{a}	1.94±0.56^{a}	2.50±1.26^{ab}

ST-6 h & ST-24 h = S. Typhimurium at 6 h and 24 h post-infection; SG-6 h & SG-24 h = S. Gallinarum at 6 h and 24 h post-infection

Supplementary Table 4. Fold change expression of cytokines and chemokines mRNA (Mean ± S.E.) following infection with *S*. Gallinarum and *S*. Typhimurium at 6 and 24 h post-infection. Different superscripts (a,b,c) across the columns denotes significant difference.

**Cytokines and Chemokines**	**ST-6h**	**SG-6h**	**ST-24h**	**SG-24h**
IL-1β	444.6±0.26^{b}	636.1±0.46^{b}	17.7±0.43^{a}	33.90±0.38^{a}
IL-6	11.55±0.39^{b}	26.9±0.21^{b}	2.1±0.59^{a}	13.39±0.55^{b}
TNF-α	21.8±0.17^{c}	0.2±0.02^{a}	0.31±0.029^{a}	4.18±0.47^{b}
IFN-γ	1.74±0.36^{b}	0.005±0.003	0.003±0.006^{a}	1.06±0.47^{b}
IL-12	8.45±0.27^{b}	0.16±0.067^{a}	0.43±0.06^{a}	3.48±0.33^{b}
IL-10	9.96±0.22^{b}	0.39±0.16^{a}	3.26±1.06^{b}	11.55±0.36^{b}
CXCLi1	18.37±0.72^{b}	0.69±0.068^{a}	7.12±1.18^{ab}	17.79±0.31^{b}
CXCLi2	1.15±0.51^{a}	7.31±0.17^{b}	0.45±0.19^{a}	1.24±0.53^{a}
IL-4	85.42±0.25^{c}	1.33±0.05^{a}	6.71±0.21^{b}	7.88±0.43^{b}

ST-6 h & ST-24 h = S. Typhimurium at 6 h and 24 h post-infection; SG-6 h & SG-24 h = S. Gallinarum at 6 h and 24 h post-infection

Supplementary Fig. 1. Intracellular survival and cytotoxicity of *S*. Gallinarum and *S*. Typhimurium in chicken dendritic cells (chMoDCs) at 3h and 48h post-infection. The correlation plot for intracellular bacterial survival and cytotoxicity; a) scattered plot matrix for *S*. Typhimurium (ST), b) scattered plot matrix for *S*. Gallinarum (SG), c) Scattered plot showing significant positive correlation between intracellular survival and cytotoxicity for *S*. Gallinarum (SG) at 48h post-infection (**p* < 0.05).

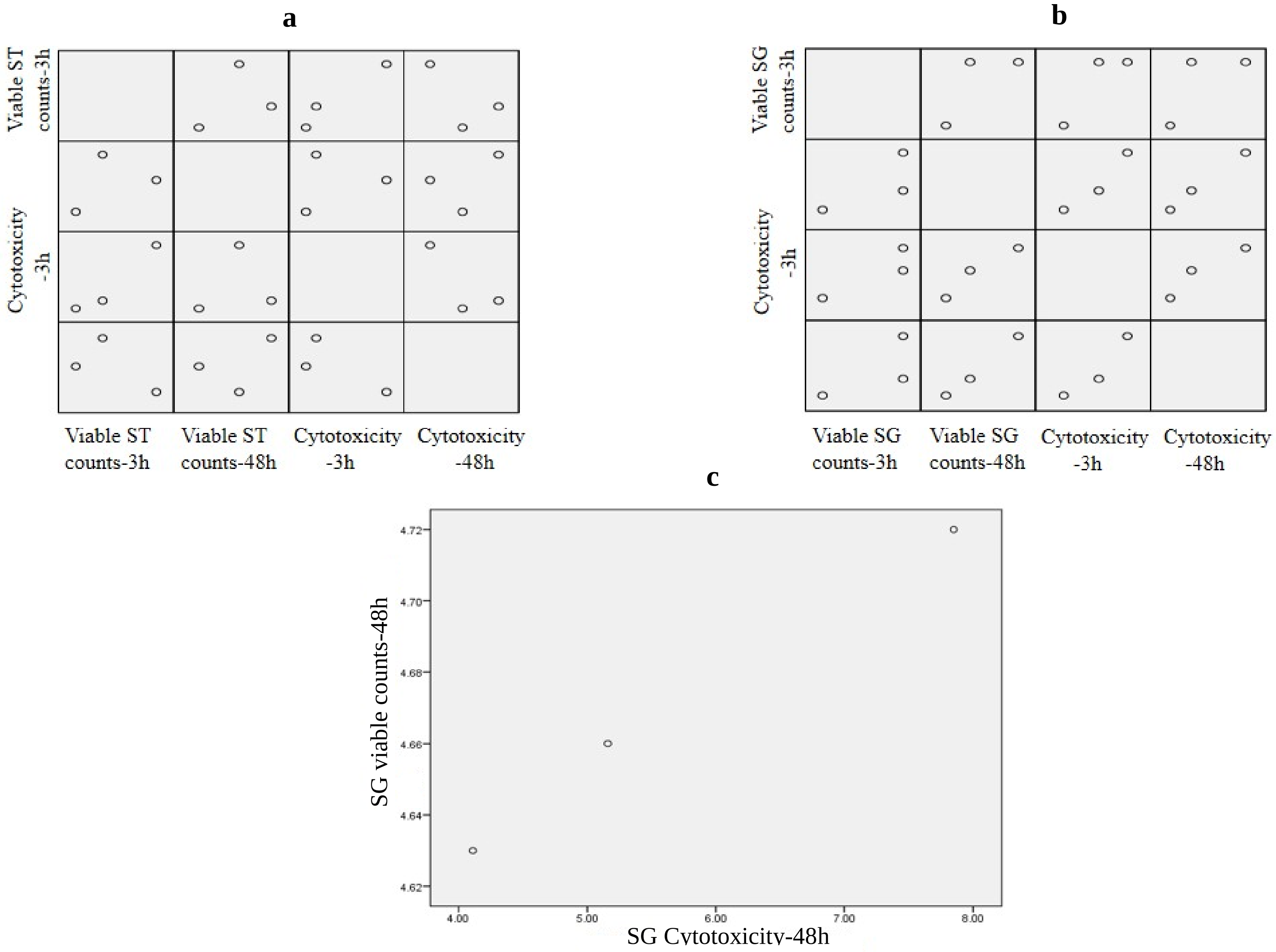

Supplement: Supplementary file 1 — Supplementary Information. [file 41598_2021_96527_MOESM1_ESM.pdf]
